# Role of Impulsivity in Explaining Social Gradient in Youth Tobacco Use Initiation: Does Race Matter?

**DOI:** 10.31586/ojn.2024.1052

**Published:** 2024-08-27

**Authors:** Shervin Assari, Payam Sheikhattari

**Affiliations:** 1 Department of Internal Medicine, Charles R. Drew University of Medicine and Science, Los Angeles, CA, United States; 2 Department of Urban Public Health, Charles R. Drew University of Medicine and Science, Los Angeles, CA, United States; 3 Marginalization-Related Diminished Returns (MDRs) Center, Los Angeles, CA, United States; 4 The Prevention Sciences Research Center, School of Community Health and Policy, Morgan State University, Baltimore, MD, USA; 5 Department of Behavioral Health Science, School of Community Health and Policy, Morgan State University, Baltimore, MD, USA

**Keywords:** Impulsivity, Tobacco Use, Substance Use, Socioeconomic Status (SES), Racial Disparities, Youth Health Disparities

## Abstract

**Background:**

Socioeconomic status (SES) is traditionally viewed as a protective factor against impulsivity and subsequent tobacco use in youth. The prevailing model suggests that higher SES is associated with lower impulsivity, which in turn reduces the likelihood of future tobacco use. However, this pathway may not hold uniformly across racial groups due to differences in impulsivity and the phenomenon of Minorities' Diminished Returns (MDRs), where the protective effects of SES, such as educational attainment, tend to be weaker or even reversed for Black youth compared to their White counterparts.

**Objectives:**

This study aims to examine the racial heterogeneity in the pathway from childhood SES to impulsivity and subsequent tobacco use initiation during adolescence, focusing on differences between Black and White youth.

**Methods:**

Data were drawn from the Adolescent Brain Cognitive Development (ABCD) Study, which includes a diverse sample of youth aged 9 to 16 years. The analysis examined the relationship between baseline family SES (age 9), impulsivity (age 9), and subsequent tobacco use (ages 9 to 16). Impulsivity was measured using the Urgency, Premeditation (lack of), Perseverance (lack of), Sensation Seeking, and Positive Urgency Impulsive Behavior Scale (UPPS-P). Structural equation modeling (SEM) was employed, with analyses stratified by race to explore potential differences in these associations.

**Results:**

Overall, 6,161 non-Latino White and 1,775 non-Latino Black adolescents entered our analysis. In the full sample, higher family SES was linked to lower childhood impulsivity and, consequently, less tobacco uses in adolescence. However, racial differences emerged upon stratification. Among White youth, higher SES was associated with lower impulsivity, leading to reduced tobacco use, consistent with the expected model. In contrast, among Black youth, higher SES was not associated with lower impulsivity, thereby disrupting the protective effect of SES on tobacco use through this pathway. These findings suggest that racial heterogeneity exists in the SES-impulsivity-tobacco use pathway, aligning with the MDRs framework, which highlights how structural factors may weaken the protective effects of high SES among Black youth.

**Conclusions:**

These findings underscore the importance of considering racial heterogeneity in the relationships between SES, impulsivity, and tobacco use. The observed disparities suggest a need for targeted interventions that address the unique challenges faced by Black youth, who may not experience the same protective benefits of high SES as their White peers. These results carry significant implications for public health strategies aimed at reducing tobacco use in racially diverse populations.

## 1. Introduction

Socioeconomic status (SES) is widely recognized as a protective factor against substance use, including tobacco, among youth [1-5]. Generally, higher SES is associated with increased parental engagement, access to better environments, lower exposure to stress, improved educational opportunities, greater parental monitoring, and association with low-risk peers [6-12]. These factors collectively contribute to lower rates of tobacco use among youth from higher SES backgrounds.

It is plausible that lower impulsivity serves as a mechanism linking high family SES to a reduced likelihood of tobacco use in youth [13-16]. Impulsivity, characterized by a tendency to act without forethought, is shaped by various environmental and developmental factors, many of which are influenced by SES. Youth from higher SES backgrounds typically have better emotion regulation, reflecting lower impulsivity and higher inhibitory control, thanks to access to resources and environments that support emotional and cognitive development. Conversely, lower SES environments may expose youth to chronic stress, limited opportunities for emotional development, and fewer supportive resources, all of which can exacerbate impulsivity. Since impulsivity is a well-established risk factor for substance use, including tobacco, it can act as a mediator in the pathway from family SES to youth tobacco use. In this context, changes in SES are linked to the likelihood of tobacco initiation and use both directly and indirectly through their impact on impulsivity, making SES an essential factor in understanding the broader relationship between SES and substance use. If impulsivity indeed mediates tobacco use among low SES youth, it offers valuable insights for developing universal interventions that address the specific needs of youth across SES levels to reduce substance use rates in vulnerable populations. However, if these pathways do not operate similarly across racial groups, there is a need for targeted and tailored, rather than universal, approaches [13-16].

Although it is well-established that higher socioeconomic status (SES) can lead to lower impulsivity and subsequently reduced tobacco use [13-16], emerging evidence suggests that this pathway may not function uniformly across racial groups [13-15,17,18]. Multiple studies have explored how contextual factors influence these associations [19-27]. However, research focused on Black populations remains limited [28,29]. Specifically, among Black youth, the protective effects of high SES on impulsivity and tobacco use tend to be weaker or disrupted, which aligns with the theory of Minorities' Diminished Returns (MDRs) [30,31].

The concept of MDRs posits that the benefits of socioeconomic resources, such as education and income, are less pronounced for Black individuals compared to their White counterparts [30,31]. In the context of impulsivity, while high SES is generally associated with lower levels of impulsivity among White youth [13-16], this protective effect is often diminished or even reversed among Black youth [13-15,17,18]. That is, the typical SES-impulsivity protective link may be disrupted for this group and high SES Black boys exhibit unexpectedly high levels of impulsivity [13-15,17,18].

Similarly, the protective link between SES and lower tobacco use may also be compromised among Black youth [32]. Despite their socioeconomic advantages, Black youth from highly educated families have been found to engage in higher rates of substance use, including tobacco, compared to their White peers [32]. This trend is not confined to youth but extends into adulthood and even older age, reflecting a broader pattern of weakened SES-substance use associations among Black individuals.

MDRs highlight the role of structural racism in undermining the impact of socioeconomic resources for Black individuals [30,31]. The disparities in outcomes between Black and White populations, despite similar SES levels, underscore the need for a nuanced understanding of how race modifies the SES-health-behavior relationship. While positive outcomes such as health and well-being are more closely tied to SES for Whites, this connection is often weaker or entirely absent for Blacks [30,31].

## 2. Aims

Given this context, the present study aims to explore the heterogeneity in the associations between SES, impulsivity, and tobacco use among youth, with a particular focus on racial differences. Using data from the Adolescent Brain Cognitive Development (ABCD) Study [33-35], which includes a diverse sample of youth aged 9 to 16, we will examine the pathway from baseline SES to impulsivity and subsequent tobacco use. We hypothesize that while the overall model may hold true for the entire sample, significant variations will exist when stratified by race. Specifically, we anticipate that the protective effects of high SES on impulsivity and tobacco use will be more evident among White youth, whereas these associations will be weaker or disrupted among Black youth, consistent with the MDRs framework [30,31].

## 3. Methods

### 3.1. ABCD Study Overview

The Adolescent Brain Cognitive Development (ABCD) [33-35] Study is a large-scale, longitudinal study designed to track the biological and behavioral development of children across the United States. Initiated in 2016, the study follows over 11,000 children recruited from 21 research sites nationwide. The ABCD Study collects a wide range of data, including neuroimaging, cognitive assessments, psychological evaluations, and environmental factors, with the goal of understanding how childhood experiences impact brain development and related outcomes [33-35].

### 3.2. Sample and Sampling

The ABCD Study employed a multi-stage probability sampling design to ensure a diverse and representative sample of U.S. youth. Participants were recruited from schools across various regions, reflecting the racial, ethnic, and socioeconomic diversity of the U.S. population. The initial cohort included children aged 9-10 years at baseline, with continued follow-up planned into adolescence and beyond.

### 3.3. Analytical Sample

For this analysis, we utilized data from the ABCD Study's baseline assessment and subsequent follow-ups through age 16. The analytical sample included youth who self-identified as either non-Latino Black or non-Latino White. Participants with incomplete data on key measures, such as SES, impulsivity, or tobacco use, were excluded from the analysis. Overall, 6,161 non-Latino White and 1,775 non-Latino Black adolescents entered our analysis.

### 3.4. Measures

#### Socioeconomic Status (Independent Variable):

Socioeconomic status (SES) was assessed using a composite score derived through principal component analysis (PCA) of several key variables. These variables included parental education, household income, family structure, and financial difficulties. Parental education was measured as the highest level of education attained by either parent and was treated as a continuous variable. Higher SES scores generated from the PCA indicated greater socioeconomic resources within the family context. Using PCA [36-38] to assess socioeconomic status (SES) offers several advantages. PCA allows for the integration of multiple SES-related variables, such as parental education, household income, family structure, and financial difficulties, into a single composite score that captures the overall socioeconomic context. This approach reduces the dimensionality of the data, effectively summarizing the most relevant SES information without losing significant variance. Additionally, PCA helps to mitigate the issue of multicollinearity among SES indicators, ensuring that the composite score represents a more robust and reliable measure of SES. By weighting the variables according to their contribution to the overall SES construct, PCA provides a nuanced and comprehensive measure that can better capture the complex and multifaceted nature of socioeconomic status, making it a powerful tool for examining its effects on various health and behavioral outcomes [39-44].

#### Impulsivity (Mediator):

Impulsivity was assessed using the Urgency, Premeditation (lack of), Perseverance (lack of), Sensation Seeking, Positive Urgency Impulsive Behavior Scale (UPPS-P) [45-53]. Higher scores indicated higher levels of impulsivity [54-62].

#### Tobacco Use Over Time (Outcome):

Tobacco use was measured at baseline and at subsequent follow-ups through age 16. Participants were asked about their lifetime, past-year, and current use of tobacco products, including cigarettes, cigars, and e-cigarettes. The primary outcome variable was any tobacco use during the study period.

#### Age and Sex (Confounders):

Age and sex were included as confounding variables in the analysis due to their potential influence on both impulsivity and tobacco use. Age was measured in years, and sex was coded as male or female.

#### Race (Moderator):

Race was treated as a moderating variable to examine whether the associations between SES, impulsivity, and tobacco use differed between Black and White youth. Race was self-reported by participants and categorized as non-Latino Black or non-Latino White.

### 3.5. Analysis

Data were analyzed using structural equation modeling (SEM) to examine the direct and indirect pathways from SES to impulsivity and tobacco use, with race as a moderator. The SEM approach allowed for the simultaneous estimation of multiple relationships and the testing of mediation and moderation effects. The analysis first tested the overall model for the entire sample, followed by stratified models to explore racial differences in the SES-impulsivity-tobacco use pathway. All models controlled for age and sex.

### 3.6. Ethics

The ABCD Study received approval from the Institutional Review Board (IRB) at the University of California, San Diego (UCSD), as well as IRBs at all other participating research sites. Prior to participation, written informed consent was obtained from all parents or legal guardians of the participants, and assent was obtained from the children themselves. The current analysis utilized de-identified secondary data from the ABCD Study to ensure participant confidentiality and privacy, in compliance with ethical guidelines for secondary data analysis. All procedures followed the principles outlined in the Declaration of Helsinki and adhered to the ethical standards of the American Psychological Association (APA) and other relevant bodies. The use of secondary data for this study was exempt from the full IRB review.

## 4. Results

Overall, 6,161 non-Latino White and 1,775 non-Latino Black adolescents entered our analysis. Non-Latino Black youth were from families with lower socioeconomic status (SES) than non-Latino White youth. However, non-Laino Black and non-Latino White youth did not differ in age and sex. All our SEM models demonstrated good fit, indicated by a Root Mean Square Error of Approximation (RMSEA) of less than 0.06 and a Comparative Fit Index (CFI) greater than 0.90. We did not rely on the Chi-square test as a marker of fit due to the large sample size, which can lead to significant Chi-square values not necessarily reflective of poor fit but rather a result of the large sample size.

**Figure 1.**
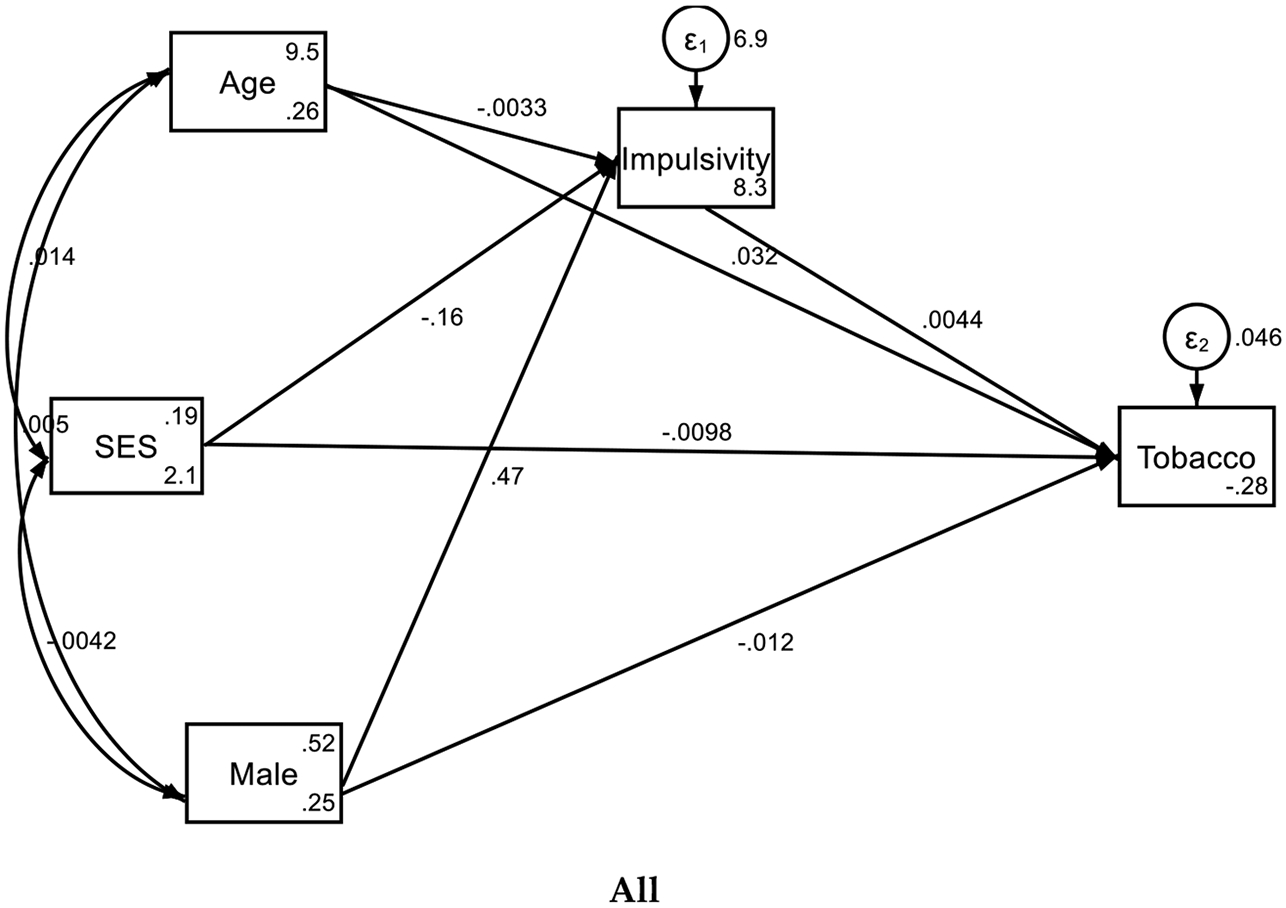

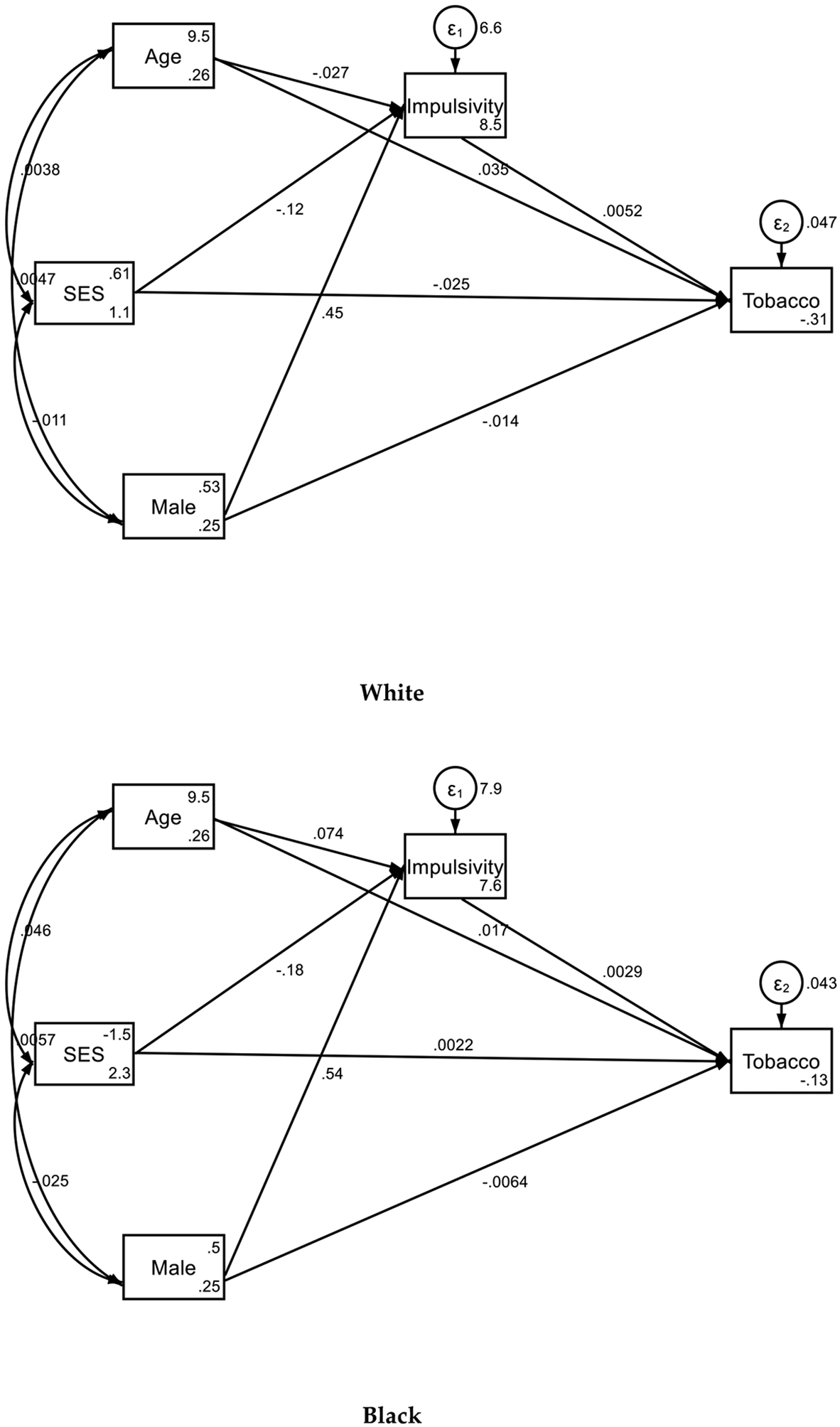
Summary of SEMs in overall, White, and Black youth

As shown in Figure 1 and Table 1, in the overall sample, baseline socioeconomic status (SES) was negatively associated with impulsivity (β = −0.157, p < 0.001), indicating that lower SES was linked to higher impulsivity levels. However, baseline age showed no significant association with impulsivity (β = −0.003, p = 0.960). Being male was significantly associated with higher impulsivity (β = 0.470, p < 0.001). The intercept for impulsivity was significant, suggesting an average baseline level of impulsivity (β = 8.349, p < 0.001).

When examining subsequent tobacco use, baseline impulsivity was positively associated with tobacco use (β = 0.004, p < 0.001). SES at baseline also showed a negative association with subsequent tobacco use (β = −0.010, p < 0.001), while age was positively associated with tobacco use (β = 0.032, p < 0.001). Being male was associated with a decrease in subsequent tobacco use (β = −0.012, p = 0.016). The intercept for tobacco use was significant, indicating a baseline level of tobacco use (β = −0.280, p < 0.001).

For White youth, baseline SES was negatively associated with impulsivity (β = −0.119, p = 0.001), suggesting that lower SES was related to higher impulsivity. Age at baseline did not show a significant association with impulsivity (β = −0.027, p = 0.718), but being male was significantly linked to higher impulsivity (β = 0.450, p < 0.001). The intercept for impulsivity was significant (β = 8.544, p < 0.001).

Regarding subsequent tobacco use in White youth, baseline impulsivity was positively associated with tobacco use (β = 0.005, p < 0.001). SES at baseline also showed a negative association with tobacco use (β = −0.025, p < 0.001), while age was positively associated with tobacco use (β = 0.035, p < 0.001). Being male was linked to a reduction in subsequent tobacco use (β = −0.014, p = 0.010). The intercept for tobacco use was significant (β = −0.306, p < 0.001).

In the Black youth subgroup, baseline SES had a stronger negative association with impulsivity (β = −0.179, p < 0.001), while age did not significantly predict impulsivity (β = 0.074, p = 0.599). Being male was associated with significantly higher impulsivity (β = 0.540, p < 0.001). The intercept for impulsivity was also significant (β = 7.561, p < 0.001).

However, baseline impulsivity did not significantly predict subsequent tobacco use among Black youth (β = 0.003, p = 0.152). Similarly, SES at baseline showed no significant association with tobacco use (β = 0.002, p = 0.522), and the association between age and subsequent tobacco use was marginally significant (β = 0.017, p = 0.084). Being male did not significantly affect subsequent tobacco use (β = −0.006, p = 0.519), and the intercept for tobacco use was not significant (β = −0.134, p = 0.154).

## 5. Discussion

This study sought to explore the racialized heterogeneity in the relationships between SES, impulsivity, and tobacco use among youth, with a particular focus on understanding how these associations differ between Black and White youth. We hypothesized that while the overall model linking high SES to lower impulsivity and reduced tobacco use would hold true for the general population, significant racial differences would emerge. Specifically, we anticipated that the protective effects of high SES on impulsivity and tobacco use would be less pronounced or disrupted among Black youth, consistent with the framework of Minorities' Diminished Returns (MDRs).

The analysis revealed a complex pattern of associations that underscores the nuanced differences between White and Black youth regarding the influence of SES and impulsivity on tobacco use. Notably, the only path that consistently emerged across both racial groups was the link between high SES and lower levels of impulsivity. This finding suggests that higher SES serves as a protective factor against impulsive behaviors across racial lines, likely due to the increased resources, stability, and opportunities for selfregulation that are more accessible in higher SES environments. However, in White youth, the protective effects of high SES extended beyond impulsivity to include a direct link to lower tobacco use. This significant direct path from SES to reduced tobacco use indicates that the advantages conferred by higher SES—such as access to better education, health resources, and supportive social environments—might translate into healthier behaviors, including a lower likelihood of initiating or continuing tobacco use.

In contrast, this direct path from SES to reduced tobacco use was not significant for Black youth. This discrepancy suggests that broader social and structural factors influencing health behaviors may operate differently across racial groups. The diminished return of SES on tobacco use in Black youth highlights persistent barriers and stressors that may mitigate the protective effects of higher SES in this group, such as exposure to discrimination, limited access to health-promoting resources, and systemic inequities. Furthermore, while impulsivity was a significant predictor of subsequent tobacco use in White youth, this association did not hold for Black youth. This suggests that impulsivity, as a psychological trait, may interact differently with social and environmental contexts depending on race. For White youth, higher impulsivity appears to directly contribute to an increased likelihood of tobacco use, aligning with established theories of risk-taking and substance use. However, the absence of a significant association in Black youth could point to other overriding factors—such as community influences, cultural norms, or stressors unique to the Black experience—that modulate the relationship between impulsivity and tobacco use.

Minorities' Diminished Returns (MDRs) refer to the phenomenon where the benefits of socioeconomic resources, such as education and income, are systematically smaller for racial and ethnic minorities compared to Whites. In this study, the MDRs were evident in the weakened link between high SES and lower impulsivity among Black youth. While high SES typically serves as a buffer against mental health disorders, this effect was significantly diminished for Black youth, leading to higher rates of impulsivity despite their socioeconomic advantages. This disruption in the SES-impulsivity link subsequently influenced the association between SES and tobacco use, where the expected protective effect was also diminished among Black youth.

The observed MDRs in this study likely stem from broader structural factors rooted in systemic racism. Despite socioeconomic gains, Black families often face persistent exposure to racial discrimination, residential segregation, and economic instability, which can negate the expected benefits of high SES. These structural barriers contribute to chronic stress, increased vulnerability to mental health issues, and a greater likelihood of engaging in substance use as a coping mechanism. The structural racism embedded in various social institutions perpetuates these disparities, leading to the diminished returns of SES for Black youth.

One of the most striking findings was that tobacco use was unrelated to impulsivity among Black youth, in sharp contrast to the protective effects of low impulsivity on tobacco use observed in White youth. This pattern suggests that the benefits of emotion control, such as reduced impulsivity, may vary significantly across racial groups. For Black youth, environmental pressures and stressors may overshadow the effects of emotion regulation, meaning that even with better emotion control, tobacco use may not decrease. In contrast, for White youth, better emotion regulation appears to directly contribute to lower tobacco use. This finding is another example of the Minorities' Diminished Returns (MDRs), where the protective effects of a psychological asset, such as low impulsivity, have weaker or non-existent impacts on health outcomes for Black youth compared to their White counterparts.

The observation that tobacco use appears to be more common among high SES Black individuals can be further explained by the concept of MDRs, which suggests that the protective effects of higher SES are often less pronounced for Black individuals compared to their White counterparts. For high SES Black individuals, the stressors associated with navigating predominantly White professional environments, experiences of racial discrimination, and the pressure to conform to social norms may contribute to higher levels of tobacco use despite their socioeconomic advantages. Additionally, the social environments and networks within which high SES Black individuals find themselves may include greater exposure to tobacco use, coupled with limited access to culturally relevant cessation resources. This pattern underscores the complexity of health behaviors within different socioeconomic and racial contexts, highlighting the need for targeted public health interventions that address the unique challenges faced by high SES Black individuals in reducing tobacco use.

The heterogeneities observed in this study highlight the pervasive impact of racism on the health and well-being of Black youth^63-65^. The disruption of the SES-impulsivity-substance use pathway among Black youth is a clear indication of how racism can undermine the protective effects of socioeconomic resources. These findings align with the broader literature on racial health disparities, which consistently shows that racism, both structural and interpersonal, plays a significant role in shaping the health outcomes of marginalized groups. Understanding these heterogeneities is crucial for developing interventions that are tailored to the specific needs and experiences of Black youth.

### 5.1. Future Research

There is a need for additional research on other marginalized and minority groups, including Asian, Native American, immigrant, and Muslim populations. Given the differences in nicotine delivery, use patterns, and regulatory aspects, the method of tobacco exposure significantly affects addiction outcomes. It is crucial to consider whether studies account for these factors, as they are essential for accurately assessing risks and designing effective interventions. Additionally, longer-term studies are needed to explore other outcomes, such as conduct, aggression, and the use of other substances.

### 5.2. Clinical Implications

The findings of this study have important clinical implications. Mental health professionals working with Black youth, particularly those from high SES backgrounds, should be aware of the unique challenges they face, including the heightened risk of impulsivity and its potential consequences. Interventions should be culturally sensitive and address the specific stressors related to racial discrimination and identity. Additionally, public health initiatives aimed at reducing tobacco use among Black youth should consider the broader context of MDRs and develop strategies that go beyond SES-based interventions to address the underlying causes of these disparities.

### 5.3. Limitations

This study has several limitations that should be acknowledged. First, the use of self-reported data may introduce biases, particularly in the reporting of sensitive issues such as mental health and substance use. Second, the cross-sectional nature of the baseline data limits the ability to establish causality between SES, impulsivity, and tobacco use. Longitudinal analyses would provide a more robust understanding of these relationships over time. Third, while the study focused on Black and White youth, the experiences of other racial and ethnic groups were not examined, which limits the generalizability of the findings. Future research should include a more diverse sample to explore the applicability of MDRs across different populations.

## 6. Conclusions

In conclusion, this study provides evidence of racialized heterogeneity in the SES-impulsivity-substance use pathway among youth, with significant implications for understanding the health disparities experienced by Black youth. The findings underscore the importance of considering race as a moderator in the relationships between SES, mental health, and substance use. The presence of MDRs among Black youth highlights the limitations of SES as a protective factor and the need for targeted interventions that address the structural causes of these disparities. Addressing the underlying racism that drives these heterogeneities is essential for achieving health equity and improving the well-being of all youth, regardless of their socioeconomic background.

## Figures and Tables

**Table 1.** Summary of SEMs in overall, White, and Black youth

			Beta	SE	95% CI		p

**All**							
SES (Baseline)	→	Impulsivity (Baseline)	−0.157	0.023	−0.203	−0.111	< 0.001
Age (Baseline)	→	Impulsivity (Baseline)	−0.003	0.066	−0.133	0.126	0.960
Sex (Male)	→	Impulsivity (Baseline)	0.470	0.067	0.338	0.601	< 0.001
Intercept	→	Impulsivity (Baseline)	8.349	0.626	7.121	9.576	< 0.001

Impulsivity (Baseline)	→	Tobacco (Subsequent)	0.004	0.001	0.002	0.007	< 0.001
SES (Baseline)	→	Tobacco (Subsequent)	−0.010	0.002	−0.013	−0.006	< 0.001
Age (Baseline)	→	Tobacco (Subsequent)	0.032	0.005	0.022	0.041	< 0.001
Sex (Male)	→	Tobacco (Subsequent)	−0.012	0.005	−0.021	−0.002	0.016
Intercept	→	Tobacco (Subsequent)	−0.280	0.046	−0.370	−0.189	< 0.001

**White**							
SES (Baseline)	→	Impulsivity (Baseline)	−0.119	0.036	−0.189	−0.049	0.001
Age (Baseline)	→	Impulsivity (Baseline)	−0.027	0.074	−0.173	0.119	0.718
Sex (Male)	→	Impulsivity (Baseline)	0.450	0.075	0.303	0.598	< 0.001
Intercept	→	Impulsivity (Baseline)	8.544	0.708	7.157	9.931	< 0.001


Impulsivity (Baseline)	→	Tobacco (Subsequent)	0.005	0.001	0.003	0.008	< 0.001
SES (Baseline)	→	Tobacco (Subsequent)	−0.025	0.003	−0.030	−0.020	< 0.001
Age (Baseline)	→	Tobacco (Subsequent)	0.035	0.005	0.025	0.046	< 0.001
Sex (Male)	→	Tobacco (Subsequent)	−0.014	0.006	−0.025	−0.003	0.010
Intercept	→	Tobacco (Subsequent)	−0.306	0.053	−0.410	−0.202	< 0.001

**Black**							
SES (Baseline)	→	Impulsivity (Baseline)	−0.179	0.051	−0.280	−0.078	< 0.001
Age (Baseline)	→	Impulsivity (Baseline)	0.074	0.140	−0.201	0.349	0.599
Sex (Male)	→	Impulsivity (Baseline)	0.540	0.144	0.258	0.822	< 0.001
Intercept	→	Impulsivity (Baseline)	7.561	1.337	4.940	10.182	< 0.001


Impulsivity (Baseline)	→	Tobacco (Subsequent)	0.003	0.002	−0.001	0.007	0.152
SES (Baseline)	→	Tobacco (Subsequent)	0.002	0.003	−0.005	0.009	0.522
Age (Baseline)	→	Tobacco (Subsequent)	0.017	0.010	−0.002	0.036	0.084
Sex (Male)	→	Tobacco (Subsequent)	−0.006	0.010	−0.026	0.013	0.519
Intercept	→	Tobacco (Subsequent)	−0.134	0.094	−0.319	0.050	0.154

SES: Socioeconomic Status
